# Triple-Endobutton and clavicular hook: A propensity score matching analysis

**DOI:** 10.1515/med-2021-0339

**Published:** 2021-09-06

**Authors:** Sujie Zhang, Haojie Zhang, Jiabo Wang, Xuehai Ma, Shaohua Gu

**Affiliations:** Department of Orthopedics, Dongzhimen Hospital Beijing University of Chinese Medicine, No. 116, Cuiping West Road, Tongzhou District, Beijing 101100, China; Department of Orthopedics, Huaian Hospital of Huaian City, Huaian City 223200, Jiang Su Province, China; Department of Orthopedics, Siyang Renci Hospital, Siyang County 223700, Jiang Su Province, China

**Keywords:** acute acromioclavicular joint dislocation, Triple-Endobutton plates, clavicular hook plate, propensity score matching, minimally invasive double-incision

## Abstract

We retrospectively analyzed the clinical data of 635 patients with acute acromioclavicular dislocation, who underwent surgery in our hospital between May 2014 and June 2020. Patients were divided into group A (clavicular hook plate) and group B (Triple-Endobutton plates via double-incision). The propensity score analysis using one to one match was performed for comparisons. We obtained 292 matched patients’ data. The matched preoperative clinical characteristics were a balance between the two groups. All clinical parameters showed insignificant differences (*P* > 0.05). Compared with group A, group B has longer operative time (*P* < 0.001) and more blood loss (*P* < 0.001); however, the mean incision length (*P* < 0.001) and length of hospitalization (*P* < 0.001) were shorter in group B than in the group A. The mean VAS in group B were significantly lower than in group A at each time point (*P* < 0.001), and the UCLA shoulder score was higher in the group B. The CMS scores were also higher in group B than in group A, including before removal and 12 weeks after removal (*P* < 0.001). The clinical efficacy of the double-incision Triple-Endobutton plate is better than the clavicular hook plate technology, and achieves anatomical reduction by reconstructing coracoclavicular ligament.

## 1 Introduction

Acute dislocation of acromioclavicular joint is one of the most common injuries, accounting for about 12% of shoulder injuries [1]. Acromioclavicular dislocation is mostly caused by direct violence, such as heavy injury or direct shoulder landing. It can also be caused by indirect violence, such as upper limb adduction when falling, and the dislocation is caused by the transmission of upper arm strength to the acromioclavicular joint. The acromioclavicular joint is composed of distal clavicle and acromion, belongs to the micro joints, and is able to move up and down, front and back, and perform rotating activity along the longitudinal axis of the clavicle. The coracoclavicular ligament of the acromioclavicular joint is in turn composed of the posteromedial cone ligament and the anterolateral trapezoid ligament [2]. Dislocated acromioclavicular joint causes joint instability, abnormal activity, “Piano-key sign” deformity, thereby causing obvious pain in shoulder joints and during shoulder joint movement, seriously affecting the work and life of patients, so the treatment of acromioclavicular joint dislocation is very important.

The clinical classification methods of acromioclavicular dislocation are varied. Rockwood classified the acromioclavicular dislocation into 6 types according to the pathological anatomical characteristics and the severity of injury [3]. The ligament of patients with IV–VI type acute acromioclavicular joint dislocation was completely broken, and second traumatic arthritis occurred easily [4]. So, most scholars suggest that surgical treatment should be performed first, but there are different opinions for treatment of acute dislocation type III. There are dozens of surgical methods reported for the treatment of acromioclavicular dislocation, each with its own advantages and disadvantages [5,6].

At present, the most widely used surgical method in clinical practice is internal fixation of clavicle hook plate, which has definite efficacy [7]. However, postoperative complications such as limited shoulder joint motion, shoulder pain, acromial impingement, and re-dislocation after internal fixation are prone to occur. In 2007, Struhl first proposed the anatomical reconstruction of the coracoclavicular ligament with Double-Endobutton for the treatment of acromioclavicular joint dislocation, and the clinical effect of short and mid-term follow-up was satisfactory [8]. On this basis, the surgeon used Triple-Endobutton technique with double incision, combined with 2 (4 strands) No. 5 sutures to anatomize and reconstruct the coracoclavicular ligament and indirectly reduce the acromioclavicular joint dislocation. Using propensity score matching (PSM) method, we assessed the clinical efficacy of the double-incision Triple-Endobutton plates and clavicular hook plate in treatment of acute acromioclavicular joint dislocation.

## 2 Materials and methods

### 2.1 Study design and population

We retrospectively analyzed the clinical data of patients with acute acromioclavicular dislocation who underwent surgery in our hospital between May 2014 and June 2020, and the patients were confirmed according to the preoperative clinical and radiographic assessment. The PSM method was performed to compare patients with Triple-Endobutton plates via double-incision to patients with clavicular hook plate. The diagnostic criteria were as follows: (1) History of trauma when the shoulder touches the ground or the patient’s arm rests on the ground. (2) The shoulder movement is restricted followed by local bruising, swelling, pain, aggravation of pain during abduction or lifting. (3) The piano-key sign is positive or acromioclavicular joint bulge and tenderness. (4) The imaging data confirmed acromioclavicular joint dislocation.

**Ethic statement:** This study was approved by the Ethics Committee of Siyang Renci Hospital. The research was performed in accordance with the World Medical Association Declaration of Helsinki, and all subjects provided written informed consent.

### 2.2 Eligibility

The criteria for the inclusion were as follows: (1) No previous history of shoulder injury and related surgery. (2) The acromioclavicular joint dislocation occurred within 2 weeks. (3) The complete clinical and follow-up data are available.

The criteria for exclusion are as follows: (1) Patients with fractures and dislocations of other parts of the body. (2) Combined with osteoporosis. (3) Combined brachial plexus nerve and blood vessel injury. (4) Patients with other major physical diseases such as liver, kidney, and malignant tumor. (5) Combined with a history of other diseases of the ipsilateral shoulder joint or surgery.

### 2.3 Operating methods and postoperative management

Patients in group A received the clavicular hook plate method and the patients in group B received the Triple-Endobutton plates via double-incision. The drainage sheet was removed within 48 h after the operation, and symptomatic treatments such as postoperative swelling and pain were routinely treated. The postoperative neck-wrist sling was suspended for 4 weeks, and the functional exercise was divided into three stages; (1) 0–3 weeks after treatment: gradually start moving the joints of the hands, wrist, elbow, and shoulder one day after the patient’s pain tolerance. Step by step functional exercises, mainly passive exercises, and shoulder pendulum exercises can be done in the first 2–3 weeks, from passive exercise to active, the range of exercise is from small to large. (2) 4–6 weeks after operation: 4 weeks after operation, the neck and wrist sling can be removed, the shoulder joint can be actively moved, and attention should be paid to avoid excessive shoulder joint movement. (3) Six weeks after surgery: Moderate total shoulder strength training, and patient can participate in outdoor exercises appropriately, but attention should be paid to avoid strenuous activities to avoid injury and dislocation again.

### 2.4 Data collection and definitions

All data were collected in a standard Excel sheet. The preoperative variables included baseline data, such as sex (male vs female), age (year), the affected side, body mass index (weight kg/height m^2^, kg/m^2^), cause of injury, Rockwood classification, and time from injury to surgery. The intraoperative variables included incision length, blood loss, operative time, and length of hospitalization.

The postoperative assessment consisted of after surgery, 7 days, 3 weeks, 6 weeks, 12 weeks, and 12 weeks after removal. The degree of pain was scored by the visual analogue scale (VAS), a total of 0–10 points, the higher the score, the more obvious the pain [9]. The UCLA (the University of California at Los Angeles) shoulder rating scales were used for upper limb function after surgery as well as the VAS time point [10]. Constant–Murley score (CMS) was used to score the function of the shoulder joint before removal and 12 weeks after surgery. The CMS includes shoulder pain (15 points), daily life (10 points, including normal work, entertainment, sleep is affected), the final height of the affected side’s hand (10 points), the active range of movement of the affected side’s shoulder joint, including front Flexion (10 points), abduction (10 points), internal rotation (10 points), and external rotation (10 points), and abduction muscle strength (25 points). Full score is 100 points [11].

The postoperative complications included coracoid fracture, re-dislocation, loss of reduction (slight and obvious), heterotopic ossification, and total complications.

### 2.5 Primary and secondary outcomes

The primary outcome was 7-days, 3-, 6-, and 12-weeks postoperative assessment score of VAS, UCLA, and CMS. The secondary outcomes were 12-week postoperative complications.

### 2.6 PSM

The PMS was used to achieve a balanced exposure between two groups at some potential confounding factors. The score calculation was obtained via the logistic regression. The matched baseline variables included sex (male vs female), age (year), the affected side, body mass index (weight kg/height m^2^, kg/m^2^), cause of injury, Rockwood classification, and time from injury to surgery. The matched ratio is 1:1 for the two groups based on the propensity score with a standard caliper width of 0.2.

### 2.7 Statistical analysis

Normally distributed continuous data were expressed as mean value ± standard deviation, and *t* test was used for comparisons between the two groups. The Mann–Whitney test was used for non-normally distributed continuous data. The ANOVA for repeated measurement was used for multiple time points comparison. The Chi-square test was used for category data. We performed the propensity score analysis using one to one match. The nearest-neighbor matching method was used with a caliper width equal to 0.2. The match algorithm was based on logistic regression. PSM matching was performed using the PSM 3.04 extension program [9]. The SPSS 23.0 was used for all analysis. *P* < 0.05 was considered significance level.

## 3 Results

### 3.1 General characteristic of study population

We finally enrolled 635 patients with acute acromioclavicular joint dislocation, including 419 (66.0%) males and 216 females (34.0%). The mean age was 42.0 ± 14.4 years. Among all patients, 150 patients received Triple-Endobutton plates via double-incision and 485 patients underwent clavicular hook plate. The surgery was performed in right shoulder for 385 (60.6%) patients and in left shoulder for 250 (39.4%) patients. The dislocation was caused by road accident in 58.3% (*n* = 370) of patients and due to fall in 41.7% (*n* = 265) of patients. According to the Rockwood classification, the types III, IV, and V were 72.3, 17.3, and 10.4%, respectively. The mean time from injury to surgery was 5.02 ± 2.3 days.

Table 1 presented the overall patient’s preoperative characteristics. Compared to patients in group A (clavicular hook plate), patients in group B (Triple-Endobutton plates via double-incision) tended to be younger (43.2 vs 38.3, *P* < 0.001). The number of right shoulder dislocation was higher in group B than in group A (68.7 vs 58.1%, *P* = 0.027). The mean BMI was also higher in group B than in group A (*P* < 0.001). About the Rockwood classification, significant difference was observed between two groups (*P* = 0.013). There were no significant differences in sex ratio and cause of injury (*P* > 0.05). No significant difference was observed in injury to surgery time (*P* = 0.501) between the two groups.

**Table 1:** Overall patient’s preoperative characteristics

Characteristics	Level	Group A (*n* = 485)	Group B (*n* = 150)	*P*
Sex (*n*, %)	Male	167 (34.4%)	49 (32.7%)	0.764
	Female	318 (65.6%)	101 (67.3%)	
Age (mean value [SD])		43.2 (15.0)	38.3 (11.6)	<0.001
The affected side (*n*, %)	Right	282 (58.1%)	103 (68.7%)	0.027
	Left	203 (41.9%)	47 (31.3%)	
BMI (mean value [SD)]		22.1 (3.0)	23.2 (3.8)	<0.001
Cause of injury (*n*, %)	Road accident	274 (56.5%)	96 (64.0%)	0.125
	Fall	211 (43.5%)	54 (36.0%)	
Rockwood (*n*, %)	III	343 (70.7%%)	116 (77.3%)	0.013
	IV	82 (16.9%)	28 (18.7%)	
	V	60 (12.4%)	6 (4.0%)	
Injury to surgery time (mean [SD])		5.0 (2.3)	5.1 (2.3)	0.501

Group A: Clavicular hook plate, Group B: Triple-Endobutton plates via double-incision.

### 3.2 Matched patient’s clinical characteristics and outcomes

To reduce the effects of data bias and confounding factors, we performed a 1:1 PSM analysis. We obtained 292 matched patients’ data (group A = 146 and group B = 146). The matched patient’s preoperative characteristics are presented in Table 2. The matched preoperative clinical characteristics were a balance between the two groups. All clinical parameters showed insignificant differences (*P* > 0.05).

**Table 2:** Matched patient’s preoperative characteristics

Characteristics	Level	Group A (*n* = 146)	Group B (*n* = 146)	*P*
Sex (*n*, %)	Male	45 (30.8%)	47 (32.2%)	0.801
	Female	101 (69.2%)	99 (67.8%)	
Age (mean value [SD])		40.2 (14.4)	38.7 (11.4)	0.309
The affected side (*n*, %)	Right	99 (67.8%)	100 (68.5%)	0.900
	Left	47 (32.2%)	46 (31.5%)	
BMI (mean value [SD])		23.0 (3.2)	23.0 (3.5)	0.916
Cause of injury (*n*, %)	Road accident	100 (68.5%)	92 (63.0%)	0.324
	Fall	46 (31.5%)	54 (37.0%)	
Rockwood (*n*, %)	III	116 (79.5%%)	112 (76.7%)	0.848
	IV	25 (17.1%)	28 (19.2%)	
	V	5 (3.4%)	6 (4.1%)	
Injury time (mean value [SD])		5.4 (2.3)	5.1(2.3)	0.347

Table 3 presented matched patient’s intraoperative characteristics between the two groups. Compared with group A, group B had longer operative time (61.9 ± 16.0 vs 75.9 ± 28.0, *P* < 0.001), and more blood loss (81.3 ± 5.4 vs 87.5 ± 2.9, *P* < 0.001). However, the mean incision length (5.9 ± 0.8 vs 10.0 ± 0.7, *P* < 0.001) and length of hospitalization (5.8 ± 2.4 vs 7.5 ± 3.4, *P* < 0.001) were shorter in group B than in the group A.

**Table 3:** Matched patient’s intraoperative characteristics between two groups

Parameters	Group A (*n* = 146)	Group B (*n* = 146)	*P*
Incision length (cm)	10.0 ± 0.7	5.9 ± 0.8	<0.001
Blood loss (mL)	81.3 ± 5.4	87.5 ± 2.9	<0.001
Operative time (min)	61.9 ± 16.0	75.9 ± 28.0	<0.001
Length of hospitalization (days)	7.5 ± 3.4	5.8 ± 2.4	<0.001

The postoperative outcomes were presented in Table 4. The mean VAS in group B were significantly lower than in group A at each time point (*P* < 0.001). The VAS of group B did not change after 6 weeks post operation. The VAS went down slowly in group A. In the time period from the completion of the operation to the time before the second operation when clavicle hook plate was removed, *P* < 0.05 which was statistically significant, indicating that the recovery effect of shoulder function after treatment was better before the second operation. However, no significant difference was observed at 12 weeks after removal, indicating that the recovery effect of the shoulder function after the new minimally invasive Triple-Endobutton plates is similar to the second operation of the clavicle hook plate in the 12th week when the hook plate is removed, but the new type of Triple-Endobutton plates is minimally invasive. In patients with Triple-Endobutton plates therapy, shoulder joint function recovers faster.

**Table 4:** Matched patient’s postoperative assessment between two groups

Parameters	Group A (*n* = 146)	Group B (*n* = 146)	*P*
VAS			
7 days	4.42 ± 1.0	3.7 ± 1.5	<0.001
3 weeks	2.1 ± 0.7	0.65 ± 0.5	<0.001
6 weeks	0.9 ± 0.7	0.5 ± 0.5	<0.001
12 weeks	1.1 ± 0.8	0.5 ± 0.5	<0.001
12 weeks after removal	0.9 ± 0.7	0.5 ± 0.5	<0.001
UCLA			
7 days	8.3 ± 1.3	13.4 ± 0.9	<0.001
3 weeks	15.9 ± 1.9	19.8 ± 1.7	<0.001
6 weeks	19.3 ± 1.6	25.6 ± 1.3	<0.001
12 weeks	26.0 ± 2.4	30.3 ± 1.5	<0.001
12 weeks after removal	30.7 ± 1.3	30.7 ± 1.4	0.896
CMS			
Before removal	84.3 ± 6.4	90.2 ± 4.0	<0.001
12 weeks after removal	87.4 ± 5.2	88.4 ± 3.9	<0.001

We further compared the CMS scores between the two groups, when the Triple-Endobutton plates therapy group was equivalent to the clavicle hook plate group without second removal, *P* < 0.05, which was statistically significant. It shows that Triple-Endobutton plates treatment of double-incision is better in the time period before the second operation of removing the clavicle hook plate. This is consistent with the UCLA shoulder score statistics for the same time period. In the two operations, *P* < 0.05 during the 12th week after the second operation when the clavicular hook plate was removed, which was statistically significant. It shows that the recovery effect of shoulder function after the Triple-Endobutton plates is better in the 12th week compared with the secondary operation of clavicular hook plate after the hook plate removal.

### 3.3 Complications

There were no significant differences in coracoid fracture, re-dislocation, and heterotopic ossification. However, the obvious loss of reduction is lower in group B than in group A (5.5 vs 13.7%, *P* = 0.017, Table 5).

**Table 5:** Matched patient’s postoperative complications between groups

Parameters	Group A (*n* = 146)	Group B (*n* = 146)	*P*
Coracoid fracture	4 (2.7%)	3 (2.1%)	0.999
Re-dislocation	4 (2.7%)	2 (1.4%)	0.951
Loss of reduction			
Slight	50 (34.2%)	42 (28.7%)	0.314
Obvious	20 (13.7%)	8 (5.5%)	0.017
Heterotopic ossification	18 (12.3%)	22 (15.1%)	0.496
Total complications	96 (65.7%)	77 (52.7%)	0.023

## 4 Discussion

The acromioclavicular joint is a micro-movement joint that participates in assisting the movement of the shoulder joint and plays a very important role in the functional activities of the shoulder joint. When Fukuda studied the anatomy and function of the acromioclavicular joint, it was found that the coracoclavicular ligament maintains the vertical stability of the acromioclavicular joint, and the conical ligament and the trapezoidal ligament are opposite in opposing forces and maintain a balance [10]. Klassen conducted research and analysis on the ligaments around the acromioclavicular joint and found that the acromioclavicular joint complex was the strongest, followed by the tapered ligament, and the trapezoid ligament the weakest [11]. The detailed anatomical structure and biomechanical research provide sufficient basis for the treatment of acromioclavicular joint dislocation that is not rigidly fixed, elastic, and more in line with anatomy and human biomechanics [12].

Previous study indicated that conservative treatment is usually used for patients with Rockwood classification I and II [10,11,12]. When the acromioclavicular joint dislocation is of Rockwood type III and above, the acromioclavicular ligament and the coracoclavicular ligament are completely broken, causing shoulder pain and restriction of activities, thereby seriously affecting the daily activities of patients [13]. Conservative treatment cannot make the acromioclavicular ligament and coracoclavicular ligament heal by themselves, and it is impossible to heal the dislocation of the acromioclavicular joint. Rolf et al reported that the early reconstruction of acromioclavicular joint injuries in type III–V avoids the inferior clinical results of delayed reconstructions using a modified Weaver–Dunn procedure after conservative treatment [14].

Therefore, surgical treatment should be performed to restore the function of coracoclavicular ligament and acromioclavicular ligament. The joints are stable in all directions, so surgery is needed to restore the anatomical structure of the acromioclavicular joint and restore the function of the acromioclavicular joint [15,16]. In the past, the clavicular hook plate was used in the treatment of acute acromioclavicular joint dislocation, and the clinical effect was satisfactory. However, as the follow-up time became longer, the complications gradually increased. Therefore, in recent years, the Triple-Endobutton plate was used for anatomy and reconstruction of the coracoclavicular ligament [17,18]. The middle suture of the acromioclavicular ligament has a safe and reliable clinical basis, and the clinical effect of short-term follow-up is satisfactory.

This study found that in terms of operation time and intraoperative blood loss, the double-incision Triple-Endobutton plate technology group was higher than that in the clavicular hook plate group, the differences were clinically statistically significant (*P* < 0.05). The reasons can be explained as follows: (1) Double-incision Triple-Endobutton surgery requires two Kirschner wires to temporarily fix the acromioclavicular joint and accurately locate the small plate. During the operation, the base of the coracoid process needs to be fully exposed, and the electric drill needs to be carefully used when drilling holes, not too fast, so as not to damage the subclavian nerves and blood vessels and drill into the chest cavity. There are more steps in the operation process than the clavicle hook plate. (2) As a new surgical method, the selected cases are before the turning point of the learning curve, their learning curve is longer, the surgeon is not experienced enough, and the operation may be unskilled, so the operation time is longer. (3) Due to the long operation time and prolonged exposure, it is necessary to fully separate the coracoid process and expose the acromioclavicular joint, which requires high surgical skills and is challenging for the surgeon, so the amount of intraoperative bleeding is large.

Postoperative shoulder joint function assessment: The excellent and good ratings of the Triple-Endobutton technique group was higher than that of the clavicle hook plate group, which may be related to the fact that the double-incision Triple-Endobutton technique is more biomechanical and non-firm fixation. Analysis of the reasons: (1) The hook part of the clavicle hook plate is inserted outside the subacromial joint, which can easily damage the subacromial capsule and form bursitis. Chronic inflammation of the soft tissue around the shoulder joint capsule causes shoulder joint pain and discomfort; (2) After the hook of the clavicle hook plate is inserted under the acromion, it destroys the normal anatomical structure under the acromion and makes the subacromial space smaller. When the shoulder joint is abducted, the greater tuberosity of the humerus is easily connected with the acromion and hook. When an impact occurs, the acromion impingement syndrome is formed, causing pain when the shoulder joint is moved, which prevents the patient from moving the shoulder joint and restricts its movement; (3) The hook plate is poorly pre-bent and does not fit on the clavicle surface. The hook plate is hooked. Excessive curvature can cause uneven stress distribution in the acromioclavicular joint, and it is easy to cause cumulative damage when the shoulder joint moves, causing pain and discomfort; (4) Due to shoulder joint pain, the patient’s affected side shoulder joint activity is reduced, functional exercise is lessened, and unable to complete daily activities and work normally, resulting in shoulder joint stiffness and a vicious circle. The Triple-Endobutton plate does not need to fix the acromioclavicular joint. The plate only contacts the clavicle and coracoid process, which can reconstruct the coracoclavicular ligament to achieve an anatomical reduction without affecting normal shoulder joint movement.

In the case of internal fixation removal, the Triple-Endobutton technology group does not need to be removed if there are no special complications after the operation. The patients in the clavicular hook plate group basically require a second operation after recovery. The reason could be: the Triple-Endobutton plate is a small plate and light weight, placed on the outside of the clavicle and under the coracoid process. Three small plates simulate the start and end points of the conical ligament and the trapezoidal ligament [19]. When the acromioclavicular joint moves, the force of the acromioclavicular joint is distributed along the plate, and the stress is evenly distributed. The loop ring is not easy to wear and cut. Biomechanical studies have confirmed that its strength and rigidity are higher than that of its own ligament, and it conforms to the anatomical structure of the coracoclavicular ligament. The material has good biocompatibility with human tissues, and will not produce foreign bodies and discomfort. There is no need to remove the steel plate through a second operation, so that the acromioclavicular joint does not need to be traumatized again, the shoulder joint function recovers better after the operation, the patient satisfaction is also higher, and the pain and economic burden of the patient are reduced. The complications proportion is higher in group A than in group B. The reason could be that one patient may have more than one complication. The short-observed time could be another reason.

This study has some limitations: First of all, this study is a retrospective study, and the risks of selection bias, implementation bias, and measurement bias may exist. Second, the collection of case data is from the same hospital. There is no multi-center hospital survey in different regions, the number of cases in this study is small, and there are certain limitations in the authenticity and comprehensiveness of the statistical results. Finally, the evaluation of postoperative efficacy of patients includes medical history inquiry, physical examination, imaging review, and other assessment scales for shoulder joint function scores. This study cannot be exhaustive, and there are limitations in evaluation indicators.

Using a PSM, our study indicates that the clinical efficacy of the double-incision Triple-Endobutton plate is better than that of the clavicular hook plate technology, and the double-incision Triple-Endobutton plate achieves anatomical reduction by reconstructing the coracoclavicular ligament, which is elastic fixation and has lower postoperative complications. Therefore, it is a recommended surgical method.

## Abbreviations

CMS: Constant–Murley score
PSM: Propensity score matching
VAS: Visual analogue scale
UCLA: University of California at Los Angeles shoulder rating scale

## References

[1] Kennedy MI, Peebles LA, Provencher MT, LaPrade RF. Acromioclavicular and coracoclavicular ligament reconstruction for acromioclavicular joint instability. JBJS Essent Surg Tech. 2019;9:4. 10.2106/JBJS.ST.18.00088 PMC6974305 32051774

[2] Flores DV, Goes PK, Gomez CM, Umpire DF, Pathria MN. Imaging of the acromioclavicular joint: anatomy, function, pathologic features, and treatment. Radiographics. 2020;40(5):1355–82. 10.1148/rg.2020200039 32762593

[3] Rockwood CJ. Fracture classification systems: do they work and are they useful? J Bone Joint Surg Am. 1994;76(5):790. 8018176

[4] Ozaki J, Kawamura I. “Zero-position” functional shoulder orthosis. Prosthet Orthot Int. 1984;8(3):139–42. 10.3109/03093648409146075 6522255

[5] Epstein D, Day M, Rokito A. Current concepts in the surgical management of acromioclavicular joint injuries. Bull NYU Hosp Jt Dis. 2012;70(1):11–24. 22894691

[6] Johansen JA, Grutter PW, McFarland EG, Petersen SA. Acromioclavicular joint injuries: indications for treatment and treatment options. J Shoulder Elbow Surg. 2011;20(2 Suppl):S70–82. 10.1016/j.jse.2010.10.030 21195634

[7] Wang MY, Jiang CY. Treatment for the shoulder joint injury. Zhonghua Wai Ke Za Zhi. 2007;45(20):1369–71. 18241582

[8] Struhl S. Double Endobutton technique for repair of complete acromioclavicular joint dislocations. Tech Shoulder Elb Surg. 2007;4(8):175–79.

[9] Kane LT, Fang T, Galetta MS, Goyal D, Nicholson KJ, Kepler CK, et al. Propensity score matching: a statistical method. Clin Spine Surg. 2020;33(3):120–2. 10.1097/BSD.0000000000000932 31913173

[10] Verstift DE, Kilsdonk ID, van Wier MF, Haverlag R, van den Bekerom M. Long-term outcome after nonoperative treatment for rockwood I and II acromioclavicular joint injuries. Am J Sports Med. 2021;49(3):757–63. 10.1177/0363546520981993 33439041

[11] North AS. Rockwood grade I and II acromioclavicular injuries: as benign as commonly believed? Joints. 2016;4(3):171–3. 10.11138/jts/2016.4.3.171 PMC5115242 27900310

[12] Mikek M. Long-term shoulder function after type I and II acromioclavicular joint disruption. Am J Sports Med. 2008;36(11):2147–50. 10.1177/0363546508319047 18583520

[13] Gstettner C, Tauber M, Hitzl W, Resch H. Rockwood type III acromioclavicular dislocation: surgical versus conservative treatment. J Shoulder Elb Surg. 2008;17(2):220–5. 10.1016/j.jse.2007.07.017 18249565

[14] Rolf O, Hann VWA, Ewers A, Boehm TD, Gohlke F. Acromioclavicular dislocation rockwood III–V: results of early versus delayed surgical treatment. Arch Orthop Trauma Surg. 2008;128(10):1153–7. 10.1007/s00402-007-0524-3 18038141

[15] Tang G, Zhang Y, Liu Y, Qin X, Hu J, Li X. Comparison of surgical and conservative treatment of Rockwood type-III acromioclavicular dislocation: a meta-analysis. Medicine (Baltimore). 2018;97(4):e9690. 10.1097/MD.0000000000009690 PMC5794375 29369191

[16] Calvo E, Lopez-Franco M, Arribas IM. Clinical and radiologic outcomes of surgical and conservative treatment of type III acromioclavicular joint injury. J Shoulder Elb Surg. 2006;15(3):300–5. 10.1016/j.jse.2005.10.006 16679228

[17] Steinbacher G, Sallent A, Seijas R, Boffa JM, Espinosa W, Cugat R. Clavicular hook plate for grade-III acromioclavicular dislocation. J Orthop Surg (Hong Kong). 2014;22(3):329–32. 10.1177/230949901402200312 25550012

[18] Lin HY, Wong PK, Ho WP, Chuang TY, Liao YS, Wong CC. Clavicular hook plate may induce subacromial shoulder impingement and rotator cuff lesion-dynamic sonographic evaluation. J Orthop Surg Res. 2014;9:6. 10.1186/1749-799X-9-6 PMC3922330 24502688

[19] Wu ZD, Jiang T, Wang HB, Gao F, Wu QM, Li XF. Comparison of the effect of arthroscopy assisted TightRope plate and Triple-Endobutton plate and Double Endobutton plate in the treatment of acromioclavicular dislocation]. Zhongguo Gu Shang. 2020;33(8):696–702. 10.12200/j.issn.1003-0034.2020.08.002 32875756

